# Caractéristiques de la leucémie lymphoïde chronique au Togo

**DOI:** 10.11604/pamj.2019.34.84.18752

**Published:** 2019-10-11

**Authors:** Essohana Padaro, Yao Layibo, Irénée Délagnon Messanh Kueviakoe, Kossi Agbétiafa, Hèzouwè Magnang, Nicole Delali Akossiwa Koudokpo, Koffi Mawussi, Ahoefa Vovor

**Affiliations:** 1 Service d’Hématologie, CHU Campus, Université de Lomé, Lomé, Togo; 2 Service d’Hématologie, CHU Sylvanus Olympio, Université de Lomé, Lomé, Togo; 3 Service d’Hématologie, CHU Kara, Université de Kara, Togo

**Keywords:** Leucémie lymphoïde chronique, épidémiologie, clinique, biologie, pronostic, Togo, Chronic lymphocytic leukemia, epidemiology, clinical, biology, prognosis, Togo

## Abstract

Les caractéristiques épidémiologiques, cliniques et biologiques de la leucémie lymphoïde chronique (LLC) sont peu étudiées au Togo. L'objectif de cette étude est de décrire ces caractéristiques au diagnostic. Il s'agit d'une étude rétrospective et descriptive des patients diagnostiqués au CHU Campus de janvier 1999 à décembre 2018. Durant les deux dernières décennies, 87 patients ont été vus pour LLC (20% des hémopathies malignes) avec une incidence annuelle de 4,35 nouveaux cas. L'âge moyen était de 61ans +/- 12,48 (extrêmes 17-85ans). Il y avait 55 femmes et 32 hommes (sex-ratio H/F=0,58). Cliniquement, 16 patients (18%) n'avaient aucun syndrome tumoral, 33 patients (38%) avaient une adénopathie, 62 patients (71%) une splénomégalie et 23 patients (26%) une hépatomégalie. Biologiquement les valeurs moyennes de la lymphocytose sanguine et médullaire étaient respectivement de 87188/mm^3^ (extremes 7000-481780/mm^3^) et 75,75% +/- 12,88 (extrêmes 44,5-96,5%), 65 patients (75%) avaient un taux d'hémoglobine inférieur à 10 g/dl et 20 patients (23%) avaient des plaquettes inférieures à 100 000/mm^3^. Au diagnostic, 67 patients (77%) étaient au stade C de Binet, 7 patients (8%) au stade B et 13 patients (15%) au stade A. L'étude des facteurs biologiques pronostiques montre que 66% avaient une β2-microglobuline supérieure à la normale et la lactate déshydrogénase (LDH) était supérieure à la normale dans 95% des cas. La LLC est une réalité au Togo. Il existe une prédominance féminine et un âge moyen de 61 ans. Les patients sont vus en majorité au stade C de Binet. Il a été noté une forte masse tumorale avec une augmentation de la LDH et de la β2-microglobuline. Le suivi actuel des patients nous permettra d'évaluer leur survie globale.

## Abstract (en)

The epidemiological, clinical and biological characteristics of chronic lymphocytic leukemia (CLL) are little studied in Togo. The purpose of this study was to describe these characteristics at the time of diagnosis. We conducted a retrospective and descriptive study of patients diagnosed at the University Hospital Campus from January 1999 to December 2018. Over the past two decades, 87 patients were seen for CLL (20% of patients with hematological malignancies) with an annual prevalence of 4.35 new cases. The average age of patients was 61 +/- 12,48 years (ranging from 17-85 years); 55 women and 32 men (sex ratio M/F 0.58) were enrolled. Clinically, 16 patients (18%) had no tumor syndrome, 33 patients (38%) had lymphadenopathy, 62 patients (71%) splenomegaly and 23 patients (26%) hepatomegaly. Biologically, the mean blood and medullary lymphocyte count was 87188/mm^3^ (ranging from 7000-481780/mm^3^) and 75.75% +/- 12,88 (ranging from 44,5-96,5%) respectively; 65 patients (75%) had haemoglobin less than 10g/dl and 20 patients (23%) had platelet count below 100000/mm^3^. At the time of diagnosis, 67 patients (77%) had Binet stage C, 7 patients (8%) stage B and 13 patients (15%) stage A. The study of biological prognostics factors showed that 66% of cases had β2-microglobulin level higher than normal and 95% of cases had LDH higher than normal. CLL is a reality in Togo with a predominance of women and an average age of 61 years. Most patients are seen at Binet stage C and their assessment has revealed huge tumor mass with increased LDH and β2-microglobulin. The current follow-up of these patients will enable us to assess their overall survival.

## Introduction

La leucémie lymphoïde chronique (LLC) est une hémopathie maligne chronique caractérisée par la prolifération clonale médullaire et l’accumulation dans le sang, les ganglions et la rate des cellules lymphocytaires (B dans 95%) exprimant le déterminant antigénique CD5 [1, 2]. La LLC est la plus fréquente des leucémies dans les pays occidentaux [3, 4]. L’âge médian au diagnostic varie entre 67 et 72 ans et les hommes sont plus susceptibles de développer la maladie que les femmes [5, 6].

La grande hétérogénéité clinique, caractéristique de la LLC, a justifié, depuis des décennies, la détermination de marqueurs pronostiques. On distingue les marqueurs « classiques », historiques, d’usage clinique variable et les facteurs « modernes », de découverte récente, mais d’utilisation en routine encore mal établie. Les facteurs pronostiques classiques ont été établis par les classifications clinico-biologiques de Rai et de Binet publiées respectivement en 1975 [7] et en 1981 [8] et sont toujours d’usage clinique quotidien. Elles représentent la première étape indispensable dans la décision thérapeutique [9]. Ces classifications évaluent principalement la masse tumorale, mais sont incapables de prédire la progression de la maladie chez les patients jeunes en stade précoce. Dans les années 1980, le type histologique de l’infiltration médullaire, nodulaire ou diffuse, a été identifié comme facteur pronostique [9]. Les principaux facteurs biologiques décrits dans les années 1990 sont représentés par une élévation du taux sérique de CD23 soluble, de la ß2-microglobuline, de la thymidine kinase (TK) sérique ou des LDH [10]. Actuellement, on admet les indicateurs pronostiques comme les facteurs sériques, l’état mutationnel des gènes codant pour la partie variable des chaines lourdes immunoglobulines (IgVH), certaines anomalies cytogénétiques (mutation p53 et délétion 17p), l’expression membranaire de CD38 et l’expression intracellulaire de la protéine 70 associées au zêta (ZAP-70) [2, 11, 12].

La LLC demeure une pathologie relativement rare en Afrique [13-16]. Les données portant sur les populations subsahariennes sont encore très peu nombreuses. Au Togo, la LLC représente 22% des hémopathies malignes identifiées au myélogramme soit 104 cas entre 1992 et 2013 [17]. Une autre étude au Togo [18] avait montré l’intérêt de la monochimiothérapie à base de chlorambucil et relevé les particularités de l’évolution sous ce traitement dans notre pays où l’immunophénotypage indispensable au diagnostic et les protocoles thérapeutiques de référence actuels sont inaccessibles en raison de leur coût et de leur disponibilité. Aucune étude n’a encore défini les caractéristiques cliniques et biologiques de la LLC au Togo. La présente étude a pour but de décrire les caractéristiques épidémiologiques, cliniques, biologiques avec évaluation du stade de Binet et étudier le profil de la ß2-microglobuline et de la LDH des patients atteints de LLC au Togo.

## Méthodes

Il s’agit d’une étude rétrospective et descriptive portant sur les dossiers de patients de janvier 1999 à décembre 2018 soit une période de 20 ans. Cette étude a intéressé tous les patients suivis dans l’unité d’hématologie clinique du CHU Campus de Lomé qui constitue la principale structure spécialisée où sont référées l’ensemble des hémopathies malignes soupçonnées ou étiquetées. Seuls les dossiers de patients dont le diagnostic de certitude a été posé ont été retenus. Ces dossiers contiennent les résultats de l’hémogramme, du myélogramme, du dosage de ß2-microglobuline et de la LDH. Nous avons considéré les normes [0,9 - 2,7μg/ml] pour la ß2-microglobuline et [230 - 460 UI/L] pour la LDH. Les données étudiées étaient l’épidémiologie, la symptomatologie clinique, les signes biologiques avec classification selon le stade de Binet, les résultats préliminaires du profil évolutif des patients. Le diagnostic de LLC a été retenu sur la base d’une lymphocytose médullaire supérieure à 30% et/ou avec un score de Matutes ≥ 4 chez les patients présentant une hyperlymphocytose supérieure à 4500 lymphocytes/mm^3^.

Les données ont été analysées par le logiciel R Studio version 1.1.453. Les données quantitatives ont été exprimées en moyenne +/- écart-type et extrêmes et les données qualitatives sous-forme de proportions. L’incidence hospitalière moyenne ou annuelle a été obtenue en rapportant le nombre de cas collectés à la durée de la période de l’étude. Le taux brut d’incidence annuelle a été obtenu en rapportant l’incidence hospitalière à l’effectif de la population générale (population de 2018 estimée à 7440364 habitants par projection après le recensement de 2010).

## Résultats

De janvier1999 à décembre 2018, le service d’hématologie clinique du CHU Campus a accueilli 4879 patients dont 4119 drépanocytaires tout et âge phénotype confondu. Quatre cent vingt-six (426) patients ont été admis pour hémopathie maligne dont 197 cas d’hémopathies lymphoïdes chroniques (HLC) soit 46,24% de l’ensemble des hémopathies malignes. Parmi les 197 cas d’HLC, nous avons enregistrés 87 cas LLC soit 44,16% des HLC et 20,42% de l’ensemble des hémopathies malignes avec une incidence annuelle de 4,35 nouveaux cas par an et un taux d’incidence brut de 0,058 cas pour 100 000 habitants.

**Figure 1:**
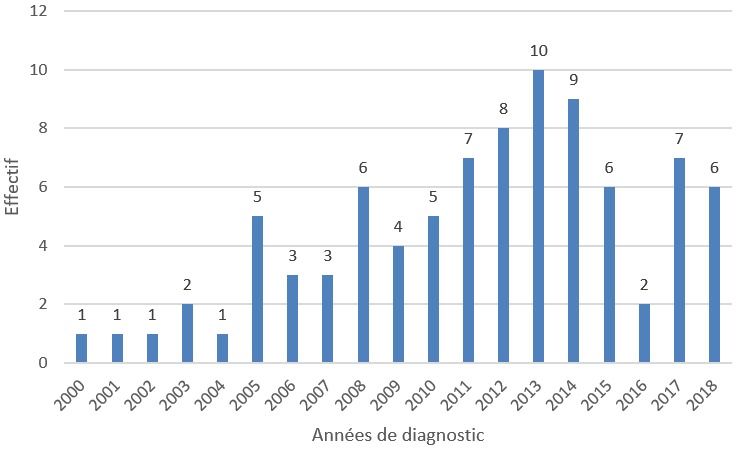
Répartition des patients selon les années de diagnostic

**Années de diagnostic:** la grande partie (46%) des diagnostics a été posée à partir de 2013 (Figure 1).

**Age et sexe:** au diagnostic, l’âge moyen des patients était de 61ans +/- 12,48 avec des extrêmes de 17 et 85ans. La tranche d’âge la plus représentée était 60 - 74 ans (Tableau 1). Il y a une prédominance féminine avec un sex-ratio H/F = 32/55 = 0,58.

**Tableau 1:** Répartition des patients selon les tranches d’âge

Tranches d’âge (ans)	Effectif	%
< 45 ans	8	9
45-59	26	30
60-74	38	44
≥ 75	15	17
Total	87	100

### Clinique

Au diagnostic, 16 patients (18%) n’avaient aucun signe clinique et leur pathologie a été découverte de façon fortuite à l’occasion d’un bilan de santé. Trente-trois patients (38%) avaient une adénopathie associée à une splénomégalie sauf chez 6 patients (7%). Toutes les aires ganglionnaires étaient atteintes soit isolément ou atteinte de plusieurs aires. La majorité des patients (49%) avaient des adénopathies de toutes les aires périphériques (Tableau 2). Soixante-deux patients (71%) avaient une splénomégalie de taille variable selon la classification de Hackett: stade I (11%), stade II (19%), stade III (36%), stade IV (16) et stade V (18%). Vingt-trois patients (26%) avaient une hépatomégalie qui était associée à une splénomégalie chez 16 patients et 4 avaient été splénectomisés avant leur admission. Seuls 3 patients avaient une hépatomégalie isolée.

**Tableau 2:** Répartition des patients selon le siège des adénopathies

	Effectif	Pourcentage (%)
Cervicales	3	9
Axillaires	4	12
Inguinales	5	15
Cervicales + Axillaires	1	3
Cervicales + Inguinales	2	6
Axillaires + Inguinales	2	6
Cervicales + Axillaires + Inguinales	16	49
Total	33	100

### Biologie

La lymphocytose sanguine moyenne était 87188/mm^3^ (extrêmes de 7000 à 481780/mm^3^). Le taux d’hémoglobine moyen était de 8,94 g/dl +/- 2,33 (extrêmes: 3,5 et 14,5 g/dl) et 65 patients (75%) avaient un taux d’hémoglobine < 10 g/dl. Le chiffre de plaquettes moyen étaient de 177655/mm^3^ +/- 96 325 (extrêmes: 16000 et 420 000/mm^3^) et 20 patients (23%) avaient des plaquettes < 100 000/mm^3^ et 18 parmi eux avaient une anémie associée. Deux patients (2%) avaient une thrombopénie sans anémie. La lymphocytose médullaire moyenne était de 75,75% +/- 12,88 (extrêmes 44,5 et 96,5 %). L’immunophénotypage avec calcul du score de Matutes a été réalisé chez 30 patients parmi les 48 nouveaux patients diagnostiqués à partir de 2012 (17 avaient un score de Matutes à 5, et 13 un score à 4). Sur le plan pronostic, 67 patients (77%) étaient au stade C de Binet, 7 patients (8%) au stade B et 13 patients (15%) au stade A. L’analyse montre que 84% des femmes sont au stade C de Binet contre 66% des hommes mais il n’existe pas de relation statistiquement significative (p=0,054) (Tableau 3). La valeur moyenne de la ß2-microglobuline était 4,16μg/ml avec des extrêmes de 0,67 et 11,6μg/ml. Les 57 patients (66%) ayant une ß2-microglobuline supérieure à 2,7μg/ml avaient toujours une LDH élevée. Pour la LDH, la valeur moyenne est de 836,4 UI/l (extrêmes 171- 5610 UI/l) et 83 patients (95%) avaient une LDH supérieure à la normale. Parmi les 67 patients au stade C de Binet, 55(82%) avaient une LDH élevée et 54(81%) avaient une ß2-microglobuline élevée. L’augmentation de ß2-microglobuline était statistiquement significative dans la survenue du syndrome de Richter (Tableau 4).

**Tableau 3:** Répartition des patients selon le stade de Binet et le sexe

	Stade A	Stade B	Stade C	Total
Féminin	6 (11%)	3(5%)	46(84%)	55(100%)
Masculin	7(22%)	4(12)	21(66%)	32(100%)

**Tableau 4:** Relation entre β2-microglobuline et LDH avec syndrome de Richter

	Evolution	Min	Max	Médian	Moyenne	ET	P
LDH	Richter	340	3441	794	1494	1269	0.201
	Non Richter	171	5614	670	770	722	
B2m	Richter	1,78	11,6	7,7	6,80	4,03	0.058
	Non Richter	0,67	10,86	3,3	3,90	2,31	

Richter = 8 Non Richter = 79. Test Mann Whitney Wilcoxon

### Devenir des patients

La majorité des patients (52%) était perdue de vue (Tableau 5). Parmi les 18 patients décédés (tous au stade C), 6 décès (33%) étaient secondaires à un syndrome de Richter, 4 décès (22%) à une septicémie, 2 décès (11%) à une insuffisance médullaire, 2 décès (11%) à une anémie décompensée puis 1 décès (6%) secondaire respectivement à un accident de la voie publique, à une hémorragie digestive, à une pneumopathie et à une cause non clairement identifiée. Huit patients ont été transférés dont 2 au Ghana, 2 en France, 2 en Belgique, 1 au Bénin et 1 au Burkina Faso. Tous ces patients avaient déclaré rejoindre leurs enfants pour les uns et les autres des proches parents pour un meilleur soutien financier et moral. Tous les patients ayant signé une décharge l’ont fait en raison des difficultés financières à honorer les soins.

**Tableau 5:** Répartition des patients selon leur devenir

	Effectif	Pourcentage (%)
Traitement en cours	9	10
Perdu de vue	45	52
Décharge	7	8
Référé	8	9
Décès	18	21
Total	87	100

## Discussion

### Limite de l’étude

Les limites de cette étude étaient liées d’abord à son caractère rétrospectif et descriptif non randomisé avec risque de biais de sélection. Il s’agit ensuite d’une étude hospitalière avec des dossiers incomplets pouvant expliquer l’effectif réduit de notre série. Enfin le plateau technique limité avec une absence des nouveaux moyens diagnostiques et pronostiques ne nous a pas permis d’établir avec exactitude le pronostic de nos patients. Néanmoins les résultats de cette étude nous permettront d’avoir une analyse de base des données sur la LLC pour des futures études mieux fournies et détaillées. Déjà sur le plan diagnostic, des efforts sont faits avec réalisation de l’immunophénotypage des lymphocytes circulants.

### Fréquence

La LLC est la plus fréquente leucémie dans les pays occidentaux [3, 4] alors qu’elle demeure relativement rare en Afrique [13-16]. En 20 ans, seulement 87 patients atteints de LLC ont été identifiés au Togo avec en moyenne 44 cas en 10 ans avec une incidence annuelle de 4,35 nouveaux cas et un taux d’incidence brut de 0,058 nouveaux cas pour 100 000 habitants. Nos données sont proches de celles de Moueleu Ngalagou au Cameroun [19] avec 57 cas en 10 ans soit 5,7 nouveaux cas par an et un taux d’incidence brut de 0,061. Elles sont inférieures aux données de Sawadogo en Côte d’Ivoire [20] avec 159 cas en 10 ans, 15,9 nouveaux cas par an et un taux d’incidence brut de 0,55. Elles sont supérieures aux données de N’golet Lo au Congo [21] avec 14 cas en 10 ans, 1,4 nouveaux cas par an et 0,07 nouveaux cas pour 100 000 habitants.

### Age-sexe

L’âge moyen de 61 ans (extrêmes: 17-85) était comparable aux études sénégalaises [13], ivoiriennes [14], nigérianes [15, 16] et éthiopiennes [22] qui ont trouvé respectivement un âge moyen de 61, 62, 60, 56 et 55 ans. Cependant, cet âge moyen au diagnostic est un peu plus élevé aux Etats Unis (72 ans) [2], en Europe notamment Angleterre (74 ans) [23] ou France (72 ans) [24]. Il y a au moins 10 ans entre l’âge d’apparition de la LLC en Afrique par rapport aux pays occidentaux. Nous pensons qu’en Afrique la LLC atteint les patients à un plus jeune âge que les patients occidentaux. Ces données peuvent conforter l’idée selon laquelle des facteurs environnementaux (restants ou non encore identifiés) pourraient être impliqués. Selon certains auteurs, la survenue précoce de la LLC en Afrique serait une conséquence du paludisme récurrent et d’autres infections, entraînant une prolifération polyclonale des cellules B qui dans une forme extrême donne une splénomégalie paludéenne hyperactive [25]. La prédominance féminine retrouvée dans notre étude et la plupart des données africaines [18-21] contraste avec les données de la littérature occidentale [5, 6]. Nous n’avons pas pu trouver d’explication plausible sauf l’espérance de vie élevée chez les femmes associée à une mortalité masculine précoce en Afrique.

### Clinique

La présentation clinique la plus habituelle de la LLC notamment les adénopathies isolées n’avait été retrouvée que chez 6 patients. En plus les adénopathies périphériques n’avaient été retrouvées que chez 33 patients (38%) et étaient associées à une splénomégalie chez 27 patients. La splénomégalie était fréquente (71% des cas). La grosse splénomégalie isolée est un mode fréquent de découverte de la LLC en Afrique [18, 26, 27].

### Diagnostic biologique

Sur le plan biologique, on notait une lymphocytose sanguine périphérique importante qui était associée à une hyperlymphocytose médullaire (> 30%). Le diagnostic de LLC était ainsi retenu avec comme argument complémentaire les caractéristiques cytologique des clones lymphocytaires malgré l’absence d’immunophénotypage des lymphocytes circulants. Ce dernier est devenu depuis 2012 l’examen qui permet de poser le diagnostic de certitude dans la plupart des cas dans notre service. Par ailleurs depuis 2012, nous avons pu poser le diagnostic de treize hyperlymphocytoses sanguines et médullaires non LLC. La difficulté réside dans son accessibilité et dans son coût pour tous les patients. En effet les prélèvements sont envoyés au laboratoire CERBA en France à un prix d’environ 100 euros qui est hors de porté de la plupart des togolais avec un salaire mensuel minimum de 50 euros. Le caryotype, la recherche du CD38, du ZAP 70, CD23, et le dosage de la thymidine kinase n’ont pu être réalisés à cause du plateau technique limité. Les patients ayant réalisé l’immunophénotypage des lymphocytes sanguins sont les premiers et les seuls patients jusqu’à ce jour à avoir bénéficié de ce bilan dans notre service.

### Stade évolutif

La majorité des patients étaient au stade C de la classification de Binet avec un taux d’hémoglobine <100 g/l chez 65 patients associée à un nombre de plaquettes <100 000/mm^3^ chez 18 patients. Ceci serait probablement lié à un retard de consultation et à un manque d’information par rapport aux centres de référence [18, 26, 27]. Les femmes se présentaient plus au stade C de Binet dans notre étude mais la significativité statistique de cette différence était insuffisante probablement du fait de l’effectif limité de notre série. Nos résultats sont équivalents à ceux de Sall *et al.* au Sénégal [13]. Par contre d’autres auteurs avaient rapporté l’influence du sexe sur le profil évolutif des patients [5]. Catovsky *et al.* [6] avaient rapporté que les signes cliniques étaient plus pauvres chez la femme que chez l’homme. Les femmes étaient plus susceptibles d’avoir le stade A de Binet que le B ou C; leur taux de survie global à 10 ans était plus grand que pour les hommes et elles avaient une meilleure réponse globale au traitement. Aucune hypothèse n’a été avancée pour expliquer cette tendance observée. Cependant, les implications du sexe sur la différence dans la pathogenèse de la LLC et son traitement nécessitent d’autres études.

### Facteurs biologiques pronostiques

Dans les pays occidentaux, comme plus de 90% des patients sont diagnostiqués au stade précoce (Rai 0, Binet A), il était devenu indispensable d’identifier des paramètres qui vont permettre de prédire l’évolution des patients afin de définir une thérapeutique adaptée [1]. Parmi eux, ce sont 1) les caractéristiques cliniques telles que l’âge, le sexe et le « performance status »; 2) les paramètres biologiques reflétant la masse tumorale ou l’activité de la maladie tels que la lymphocytose, le taux de LDH, le type d’infiltration médullaire ou le temps de dédoublement de la lymphocytose; 3) des marqueurs sériques tels le CD23 soluble, la ß2-microglobuline ou la tyrosine kinase; 4) les marqueurs génétiques des cellules tumorales tels que les anomalies cytogénétiques et génétiques, l’état mutationnel des gènes codant pour la partie variable des chaines lourdes immunoglobulines (IgVH) et/ou les marqueurs considérés comme corrélés (CD38, ZAP 70) [1]. Dans notre contexte, la majorité des patients (77%) étaient vue au stade C de Binet. La LDH et la ß2-microglobuline nous ont ainsi permis d’évaluer en priorité la masse tumorale et surtout de prédire la survenue du syndrome de Richter pour lequel nous avons enregistré 8 cas. Ce nombre est probablement sous-estimé en raison du fort taux de patients perdus de vue (52%).

La ß2-microglobuline est une protéine extracellulaire appartenant au complexe HLA de classe Keating *et al.* [10] ont montré chez 622 patients qu’une élévation de la ß2-microglobulinémie était associée à une diminution de la survie à la fois des patients traités et non traités au cours de la LLC. Dans la LLC, Hallek *et al.* [28] ont montré aussi que le taux de ß2-microglobuline et de TK représentaient deux facteurs pronostiques indépendants de diminution de survie sans progression (SSP), sur une série de 113 patients. Ces marqueurs « traditionnels » ont néanmoins permis de définir d’une part les LLC « indolentes » caractérisées par une hémoglobine supérieure à 130 g/l, une lymphocytose inférieure à 30 G/l, l’absence d’adénopathie, une infiltration médullaire non diffuse et un temps de doublement lymphocytaire supérieur à 12 mois [29] et, d’autre part, un index pronostique de survie globale (SG), récemment publié par l’équipe de MJ Keating, qui distingue trois groupes pronostiques selon l’âge, la ß2-microglobulinémie, la lymphocytose, le sexe, le stade de Rai et le nombre d’aires ganglionnaires atteintes [30].

Très récemment, Heng Li *et al.* [31] en Chine, ont montré, dans une population de patients porteurs de LLC avec délétion 17p, une diminution de la survie globale dans le groupe des patients ayant une augmentation de la LDH. Dans notre série, 57 patients avaient un taux de ß2-microglobuline élevé associé toujours à une élévation de la LDH. Le suivi actuel des patients nous permettra d’établir l’influence de ces facteurs biologiques pronostiques sur l’évolution de nos patients.

## Conclusion

La LLC, un syndrome lymphoprolifératif relativement rare constitue une réalité au Togo. Toute hyperlymphocytose sanguine chez l’adulte doit faire suspecter le diagnostic de syndrome lymphoprolifératif en particulier la leucémie lymphoïde chronique dans notre contexte. Cette étude nous a permis de définir les caractéristiques de la LLC au Togo. Il existe une prédominance féminine et l’âge moyen de survenue est de 60 ans avec un syndrome tumoral important et une lymphocytose sanguine élevée. Les patients sont vus en majorité au stade C de Binet. Il existe une forte masse tumorale avec une augmentation de la LDH et de ß2-microglobuline. La réalisation systématique chez tous les patients de l’immunophénotypage sur sang périphérique et des nouveaux paramètres pronostiques doivent nous permettre un meilleur suivi et une meilleure évaluation de la survie afin d’améliorer le pronostic.

### État des connaissances actuelles sur le sujet

- La LLC est la plus fréquente des leucémies dans les pays occidentaux. L’âge médian au diagnostic varie entre 67 et 72 ans et les hommes sont plus susceptibles de développer la maladie que les femmes;
- Les facteurs pronostiques classiques établis par les classifications clinico-biologiques de Rai et de Binet sont toujours d’usage clinique quotidien malgré l’existence de nouveaux facteurs pronostics biologiques. Elles représentent la première étape indispensable dans la décision thérapeutique;
- Au Togo, la LLC représente 22% des hémopathies malignes identifiées au myélogramme et chlorambucil, seule alternative thérapeutique, demeure toujours efficace.

### Contribution de notre étude à la connaissance

- La présente étude montre que sur le plan épidémiologique, contrairement aux données occidentales, il existe une prédominance féminine et la LLC survient chez des sujets plus jeunes au Togo comme dans la plupart des pays africains;
- Depuis 2012, l’immunophénotypage est devenu l’examen biologique pour poser le diagnostic de certitude de la LLC et a permis le diagnostic étiologique de treize hyperlymphocytoses sanguines et médullaires non LLC;
- Les patients sont vus en majorité au stade C de Binet et il existe une forte masse tumorale avec une augmentation de la LDH et de la ß2-microglobuline.

## Conflits d’intérêts

Les auteurs ne déclarent aucun conflit d'intérêts.
